# A Novel Model of *Staphylococcus aureus*-Induced Lymphoplasmacytic Rhinosinusitis in Rats

**DOI:** 10.3390/ijms25063336

**Published:** 2024-03-15

**Authors:** William Murphy, Sha Liu, Karen Hon, John Finnie, George Spyro Bouras, Sholeh Feizi, Ghais Houtak, Gohar Shaghayegh, Erich Vyskocil, Peter-John Wormald, Sarah Vreugde, Alkis J. Psaltis

**Affiliations:** 1 Department of Surgery-Otolaryngology Head and Neck Surgery, Basil Hetzel Institute for Translational Health Research, Central Adelaide Local Health Network, Woodville South, SA 5011, Australia; 2 The Department of Surgery, Faculty of Health and Medical Sciences, University of Adelaide, Adelaide, SA 5005, Australia; 3 Division of Research and Innovation, University of Adelaide, Adelaide, SA 5005, Australia; 4 Department of Otolaryngology, Head and Neck Surgery, Medical University of Vienna, 1090 Vienna, Austria

**Keywords:** *Staphylococcus aureus*, chronic rhinosinusitis, lymphoplasmacytic inflammatory response, rodent model

## Abstract

Chronic rhinosinusitis (CRS) is characterized by sinonasal mucosal inflammation. *Staphylococcus aureus (S. aureus)* is associated with severe CRS phenotypes. Different animal models have been proposed to study the association of CRS and *S. aureus*. However, current animal models are expensive due to the use of large animals, have high barriers to ethics approval, or require invasive surgical intervention, necessitating a need for a model that can overcome these limitations. This study aimed at establishing a reliable and efficient rat lymphoplasmacytic inflammatory model for rhinosinusitis. Sprague Dawley rats received a daily intranasal application of 20 μL of saline, *S. aureus* CI-182 exoprotein (250 μg/mL), or exoprotein CI-182 in combination with *S. aureus* clinical isolate (CI-908 or CI-913) 10^8^ colony-forming unit (CFU)/mL. The rats’ sinuses were harvested at 1 and 2 weeks post-intervention. The CFU and histopathologic examination of inflammation were evaluated. *S. aureus* clinical isolates CI-908 or CI-913 in combination with the exoprotein (CI-182) had higher CFUs and caused persistently higher inflammation at both the 1 and 2-week post-intervention compared to the exoprotein and saline group. The observed inflammatory cell type was lymphoplasmacytic. This study provided evidence that the combination of a *S. aureus* exoprotein with *S. aureus* induces inflammation that persists for a minimum of two weeks post-intervention. This model is the first known animal model to create the lymphoplasmacytic inflammation subtype seen in CRS patients. This offers a cost-effective, accessible, non-invasive, and easy-to-replicate model to study the causes and treatment of such inflammation.

## 1. Introduction

Chronic rhinosinusitis (CRS) is characterized by the inflammation of the mucosal lining of the paranasal sinus [1,2,3,4]. Approximately 10% of Western populations are affected by this disease [5]. Various studies have linked *S. aureus* to the CRS pathophysiology, in particular the more severe disease phenotypes [6,7]. *S. aureus* is the most frequently cultured bacteria in patients with CRS exacerbation [8] and influences inflammation by disrupting epithelial barrier function, impairing mucociliary clearance and inducing innate and adaptive immune responses, which may result in polyp formation [6]. Clinically, rhinosinusitis can be classified according to the duration of symptoms, including acute, subacute, or chronic [9,10]. Acute rhinosinusitis is typically virally mediated and lasts for 2–3 days. When symptoms persist beyond 5–7 days, secondary bacterial superinfection is thought likely and, in these patients, neutrophilic inflammation predominates [11]. CRS, on the other hand, is characterized mainly by the Th1, Th2, and Th17 inflammatory responses. Recently, focus on the understanding of CRS has shifted to endotyping, with further investigation into the underlying inflammatory types and the associated disease outcomes intensifying. On a cellular level, five phenotypes of nasal polyps have been reported, including eosinophilic, neutrophilic, lymphocytic, plasma cell, and a rarer lymphoplasmacytic predominant phenotype [12]. Attention has primarily surrounded eosinophilic driven inflammation, as it is the most prominent type observed in the Caucasian population. Little focus has been paid to the rarer mixed lymphoplasmacytic subtype. Eosinophilic driven inflammation is characterized by its responsiveness to steroids, whereas non-eosinophilic subtypes have low responsiveness to corticosteroids [13,14]. CRS with lymphoplasmacytic infiltration is associated with early polyp recurrence post-surgery that is often steroid-resistant. These patients often require long-term macrolide therapy for disease control [12]. Interestingly, patients treated with benralizumab for eosinophilic predominant CRS resulted in a reduction in eosinophilic inflammation and a shift towards lymphoplasmacytic inflammation, potentially suggesting underlying lymphoplasmacytic inflammation being present beneath eosinophilic driven inflammation, warranting further exploration of lymphoplasmacytic inflammation in CRS [15]. The current literature on CRS endotypes and biomarkers suggests that medical treatment should be tailored to the patients, including corticosteroids, antibiotics, and biologics [16]. Further research into the lymphoplasmacytic subtype is clearly needed to better understand the relationship between this inflammatory infiltration and its association with CRS to guide disease management.

Experimental research many uses animal models in the context of understanding the pathophysiology of diseases, and in preclinical studies to test the safety and effectiveness of novel therapies [17]. The development of animal models for sinusitis dates to the rabbit model developed by Hilding et al. [18]. Subsequent models have also been established in mice and sheep [19]. To date, there is no established animal sinusitis model focusing on lymphoplasmacytic inflammation. Currently, there is no perfect animal model that faithfully replicates the pathophysiology of acute or chronic rhinosinusitis, with each currently available model having their own deficiencies.

Ethical guidelines categorize research animals into two groups: small and large animals. Small animals only necessitate approval from a local animal ethics committee, while larger animals, including sheep, require approval from both the central and local animal ethics committees. Many existing sinusitis models entail invasive procedures and surgical interventions, leading to potential harm and discomfort for the animals involved [20,21].

Although mice are commonly used in research because of their low cost, ease of maintenance, and genetic modifications, their sinonasal anatomy differs substantially from human anatomy. They lack a true sinus, and their small size can limit tissue sampling, which also presents mechanistic limitations [20,22]. Furthermore, they lack essential genes, such as the cystic fibrosis transmembrane regulator (CFTR) gene, important in mucociliary clearance, limiting their use for studying the pathophysiology of CRS and other various phenotypes [22,23].

Rabbit models present certain advantages, largely based on their size and well-developed sinuses, allowing relatively easy sinus access to create inflammation and for sampling tissues. Nevertheless, there are significant costs and ethical considerations that need to be considered. These costs include dedicated housing facilities as well as the need for well-trained and skilled large animal handlers. Complications from the instrumentation of the sinuses, such as epiphora and pneumonia, are not uncommon and raise ethical concerns regarding the impact of such models on a rabbit’s quality of life [20,24].

A *S. aureus* biofilm-sheep frontal sinusitis model was established and successfully used in various preclinical safety and efficacy studies [25,26,27]. Sheep possess sinus anatomy and physiology closely resembling that of humans. Nevertheless, they are subjected to stringent ethical regulations and come with very high housing costs.

Considering the constraints outlined above regarding the currently available models, this project sought to assess the suitability of a rat model for investigating lymphoplasmacytic rhinosinusitis. By creating a rat model with rich lymphoplasmacytic infiltration, the further characterization of this rarer subtype was possible and provided a viable in vivo model for further investigation into potential underlying disease mechanisms and treatment targets associated with this inflammation type. Although still considered small animals, rats offer several advantages over mice. They are larger in size, translating into easier access to their sinuses, and unlike mice, they also possess (rudimentary) paranasal sinus cavities and an increased number of submucosal glands, which renders them more physiologically akin to humans than mice [28].

## 2. Results

### 2.1. S. aureus Clinical Isolate Selection

*Staphylococcus aureus* (*S. aureus*) clinical isolates (CI-182, CI-908, and CI-913) were obtained from patients with CRS. They were selected from patients with high CRS disease severity scores, being symptomatic (22-item sino-nasal outcome test (SNOT-22)), radiological (Lund–Mackay) and endoscopic (Lund–Kennedy) scores (Table 1), reflecting high disease burden strains. All patients (1 male and 2 female) had CRS with nasal polyps (CRSwNP) and asthma and one patient also had gastro-esophageal reflux disease (GORD) and another aspirin sensitivity.

### 2.2. Rat Nasal Cavity CFU Count

**Figure 1:**
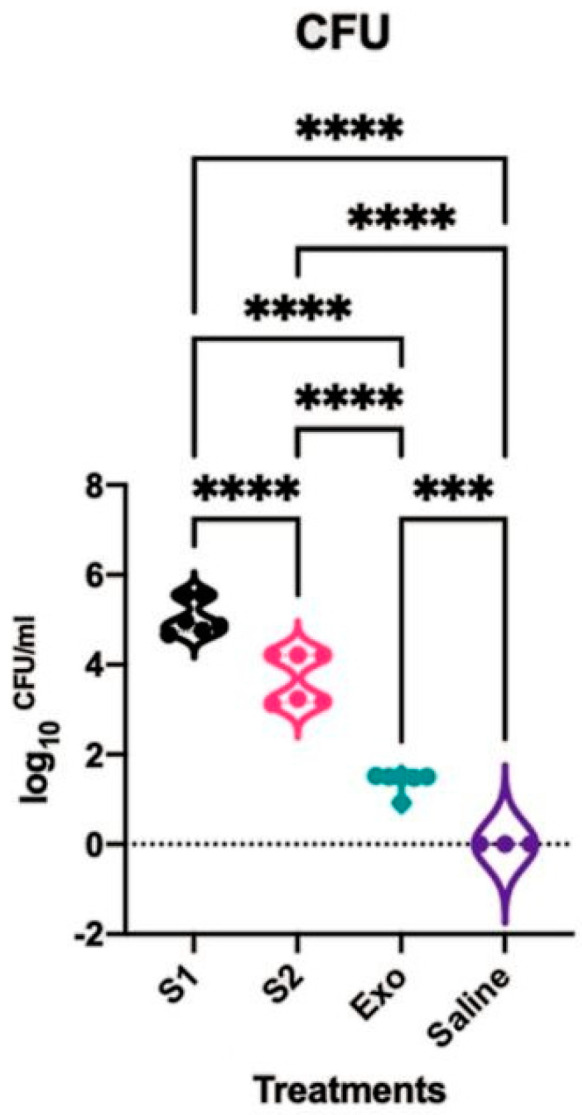
*S. aureus* CFU. The CFUs of *S. aureus* strains CI-908 (S1), CI-913 (S2), exoprotein (Exo), and the saline control in the nasal cavity were analyzed, and the significance was determined using an ANOVA. The asterisk (***) denotes statistical significance at *p* < 0.001 and **** *p* < 0.0001. *S. aureus*: *Staphylococcus aureus*; CFU: colony-forming units.

The number of colony-forming units (CFUs) of *S. aureus* harvested from both nostrils at day 37 and day 44 were investigated. The CFU results were similar between both time points and hence the datapoints were merged. The rats challenged with only exoproteins as well as with any of the CIs in the exoproteins had higher CFUs compared to the saline-treated group, which did not grow any *S. aureus* strains (*p* < 0.05). The rats challenged with CI-908 had significantly higher CFUs than those challenged with CI-913, and both were higher than the exoprotein-only-treated rats or the control group. Long-read sequencing identified *S. aureus* in the exoprotein-only-treated group as CI-913. This suggests the presence and subsequent significant difference in CFUs between the exoprotein group and the saline group, which was likely due to the cross-contamination of the exoprotein-only-treated group with CI-913 (Figure 1).

### 2.3. S. aureus Clinical Isolates and Exoproteins Induced Significant Inflammation

The inflammatory infiltrate, primarily consisting of lymphoplasmacytic cells, was prominent and infiltrated the epithelium, leading to the disruption of the lamina. Inflammation was present in both the respiratory and olfactory epithelium (Supplementary Figure S3). There was non-specific peribronchial infiltration observed in the exoprotein and both *Staphylococcus* groups. The kidney and spleen had no observable changes in any of the rats (Supplementary Figure S4).

**Figure 2:**
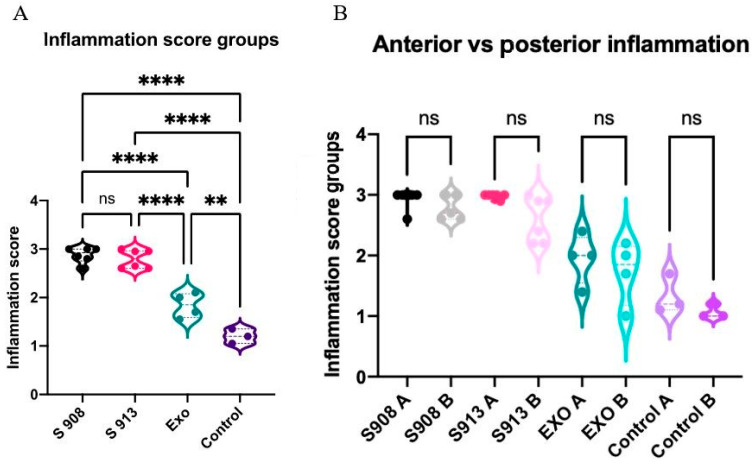
*S. aureus* and exoprotein-induced inflammation in vivo. (**A**) Inflammation scores of the sinonasal cavities for rats challenged with CI-908 (S 908), CI-913 (S 913), exoprotein or control (saline). (**B**) Anterior (**A**) and posterior sections (**B**) were compared with the samples from different segments of the same rat. S 908 n = 6; S 913 n = 6; Exo n = 4; and control n = 3. The significance was determined by comparing the results with the saline control. The asterisks indicate statistical significance (**: *p* < 0.01; ****: *p* < 0.0001), ns = not significant. *S. aureus*: *Staphylococcus aureus*.

For the assessment of inflammation, ten areas with the highest degree of inflammation were selected from each animal. The severity of lymphoplasmacytic infiltration in the lamina propria was graded by a pathologist blinded to the treatment groups on high-power (X40) fields ranging from 0 to 3, indicating no inflammation, mild, moderate, or severe inflammation, respectively. Representative images for each grade (0 to 3) are presented in Table 2. The examination revealed significant inflammation in the rats inoculated with *S. aureus* and/or the exoprotein, particularly in the *S. aureus* CI groups when compared to the exoprotein and saline control group. Additionally, the exoprotein group exhibited significantly greater inflammation than the saline control group (Figure 2A and Table 2). Inflammation was observed both anteriorly and posteriorly without any significant difference between both locations (Figure 2B).

### 2.4. S. aureus Invades the Nasal Mucosa

The results of the gram staining procedure confirmed the presence of *S*. *aureus*. This bacterium was observed to colonize the epithelial layer of the sinuses, which constitutes the outermost cellular lining of the nasal passages and was identified within epithelial cells. Further investigation revealed that *S. aureus* also possessed the capability to infiltrate the subepithelial region and breach the lamina propria, a thin layer of connective tissue located beneath the epithelium. These findings suggest that the bacterium exhibits the capacity to penetrate “damaged” nasal tissue (Table 3).

## 3. Discussion

In this study, our objective was to develop a rat sinonasal inflammation model that mimics the inflammation seen in *S. aureus* CRS in humans. This is the first animal model that reflects the inflammatory milieu of the less common and less well-described lymphoplasmacytic subtype of CRS. As the focus on CRS shifts towards endotyping and targeted treatment options, this model provides value in establishing the first in vivo model characterized by inflammation, reflective of that seen in lymphoplasmacytic predominant CRS and can be used to help further characterize its rarer inflammatory subtype. By abstaining from surgical procedures and invasive techniques, we not only prioritized the well-being and comfort of the rats but also aimed to create an easy-to-replicate inflammatory model, different to previous animal models which often require surgical interventions. We deliberately chose a rat model given its smaller size than rabbit and sheep, meaning that the animals were easier and cheaper to house, also mitigating many of the availability and ethical concerns that occur with using larger animals.

We utilized a *S. aureus* exoprotein, both individually and in conjunction with two distinct strains of *S. aureus*. The results demonstrated that both the exoprotein alone and the combination of exoprotein with the *S. aureus* strains induced notable and widespread inflammation. The combination of the exoprotein with the *S. aureus* strains demonstrated markedly elevated levels of inflammation in comparison to both the control group and the exoprotein-alone group. This suggests that although the exoprotein can in itself generate an inflammatory response, additional bacterial factors produced by live bacteria further exacerbated the inflammation. We indeed observed significantly higher CFU counts in the rats treated with both CIs of *S. aureus* compared to both the control group and the exoprotein-alone group. This finding was corroborated via gram staining, which confirmed that the inoculated *S. aureus* bacteria successfully colonized and invaded the sinonasal mucosal membrane. Even though *S. aureus* was also cultured in the exoprotein-only group, likely due to the contamination of the exoproteins by live bacteria, the CFUs were lower along with significantly reduced levels of inflammation compared to the groups treated with the clinical isolates. This suggests that the level of inflammation is indeed linked to the bacterial load. These findings align with microbiome studies that showed that the severity of CRS is directly related to an increased bacterial load [19,29,30].

The histopathological analysis revealed significant lymphoplasmacytic infiltration with goblet cell hyperplasia in both the anterior and posterior segments of the nasal tissue in the *S. aureus*-infected rats. Although there was no overall difference between the anterior and posterior regions, there was a tendency for the inflammation to be more widely distributed in the former, possibly due to the anatomical differences between these two regions, with the anterior segments receiving more consistent exposure compared to the deeper segments.

The inflammation observed was consistent with prior studies showing increased inflammation in the subepithelial layer and lamina propria of the nasal mucosa after exposure to *S. aureus* [27,31]. Such severe inflammation with goblet cell hyperplasia is also indicative of epithelial remodeling after chronic antigen exposure [32], and was observed in CRS, chronic airway diseases, cigarette smoke exposure, and cystic fibrosis [33,34,35,36]. The lymphoplasmacytic infiltrate was observed most prominently in the *S. aureus*-infected groups, consistent with the inflammatory milieu observed in CRS [10]. Although lymphoplasmacytic infiltrate is considered rarer compared to the eosinophilic subtype, Mariano et al. [37] examined 277 CRS patients and found lymphoplasmacytic infiltration to be the prominent infiltrate in 111 patients, potentially rendering it a more important inflammatory subtype in CRS than originally believed. Plasma cells (effector B cells) originate from B lymphocytes and secrete antibodies in response to antigens [38]. B cell activation and excess antibody production is associated with CRS, most prominently with CRSwNP [39,40,41] and is consistent with increased levels of plasma cells and B cells in the polyp tissue from CRSwNP patients [39,40,42,43,44,45]. T-lymphocytes (T cells) differentiate into effector T cells, such as CD4+, CD8+, regulatory, cytotoxic, or helper T cells. Different effector T cell subtypes are involved in CRSsNP and CRSwNP [46,47,48]. A meta-analysis by Shen et al. [49] showed different inflammatory infiltrates and treatment responsiveness based on the country of origin, with nasal polyps in the Asian population characterized by infiltrates more linked to type one inflammation, which exhibited a greater response to macrolides. In contrast, the Western population’s polyps are more associated with type two inflammation, which demonstrates better responsiveness to steroids. Further investigation into the lymphoplasmacytic cell subtype could improve the understanding of the pathogenic relationship between *S. aureus* and CRS, and further exploration could lead to more accurately targeted therapies tailored to this subgroup of patients. Interestingly, in our study, there was no significant difference in the extent of inflammation that was observed between the two time points taken one week apart, even though the rats did not receive further *S. aureus* during this time. Together, our findings indicate the activation of a more chronic immune response in the rats with robust lymphoplasmacytic immune cell infiltration and goblet cell hyperplasia.

The use of the exoprotein alone induced significant inflammation, suggesting the importance of secreted inflammatory proteins in the development of this response. This could be due to the effect of the exoprotein impairing mechanical barriers, such as tight junctions, which contribute to the development of CRS or possibly due to antibodies to the *S. aureus* exoprotein acting as a superantigen [44,50,51]. *S. aureus* enterotoxins (SAEs) are a well-described component of the *S. aureus* exoprotein. They can function as superantigens, resulting in the production of SAE-specific antibodies that can potentially establish sustained inflammation. Our study’s findings support this in addition to the direct disruptive effect the exoprotein has on the nasoepithelium. This breach is likely to promote the subepithelial colonization of *S. aureus* in patients with sinusitis and possibly prolongs the duration of inflammation. To minimize contamination, the exoprotein and the *S. aureus* groups were prepared separately, the saline treatment was administered before the infected groups, the rats in the saline group were kept at other ends of the room, the isoflurane box was cleaned between usages, and the room was cleaned pre-treatment and post-treatment. However, our finding of the presence of *S. aureus* in the exoprotein-only-treated group suggests cross-contamination, possibly due to the rats also being housed in the same room despite being in separate cages. The extent to which this contributed to the inflammation seen in this group cannot be discounted, forming a limitation of this study, and does suggest that further experiments mitigating contamination are required to confirm our results.

While the inflammatory environment observed in this study aligns with that observed in the context of CRS in humans, it is important to note a limitation. Although the cell types present in the inflammatory milieu mirror those seen in CRS, the designated time interval of three months of symptoms, as outlined by current guidelines for humans, was not met. As a result, future investigations should aim to determine if the inflammation persists three months post-intervention. Similarly, the inflammation milieu is only representative of that observed in CRS, inflammation is complex, and the underlaying pathology observed and factors interacting may be different in an in vivo sinonasum compared to humans. This is a limitation of this model, which hinders insight into human CRS pathology. The grouping of the 21 rats into different experimental groups resulted in a limited number of animals per group, which could potentially affect the statistical power of the findings, acting as a further limitation.

## 4. Materials and Methods

### 4.1. Animals

The animal procedures were conducted in accordance with the Australian Code for the Care and Use of Animals for Scientific Purposes and were approved by the Animal Ethics Committee of the University of Adelaide, Australia (Approval ID M-2022-059). The rats were housed under standard conditions. The lights were on for 12 h daily, and the cage temperature was 22 ± 1 °C, with continual access to water and standard regular chow and libitum. To minimize animal distress, music was provided, and the animals were handled regularly to habituate the animals to noise and being handled. The enrichment of the animal’s environment was provided through the use of cardboard boxes, rolls, and shredded paper for the animals to hide in, chew, and play with. Furthermore, daily checks for any signs of distress were conducted.

### 4.2. Staphylococcus aureus Clinical Isolates Cultured from Patients

Ethics approval for obtaining the bacterial swabs was granted by The Central Adelaide Local Health Network Human Research Ethics Committee (reference HREC/15/TWEH/132). Patients were recruited if they were undergoing endoscopic sinus surgery for chronic rhinosinusitis. The diagnostic criteria were based on the European Position Statement on CRS [9]. Written informed consent was provided by all the patients before the study commenced. The clinical histories and demographics of patients along with the severity of CRS were recorded. The disease severity was based on the completion of the Lund–Kennedy (LK), Lund–Mackay (LM), and the 22-item sino-nasal outcome test (SNOT-22) [52,53,54,55,56].

The bacteria were harvested using a Transwab (Medical Wire & Equipment, Corsham, Wiltshire, UK) after brushing gently against the middle meatus. The bacteria were then cultured on 1.5% trypticase soy agar (TSA) (Oxoid, Thebarton, SA, Australia) overnight at 37 °C. Individual colonies were re-streaked on 1.5% TSA plates and incubated overnight at 37 °C again. Individual colonies were identified using MALDI-TOF. The isolates of *S. aureus* were stored in 50% glycerol stock (Merck, Life science, Bayswater, Victoria, Australia) at −80 °C for future use.

### 4.3. Staphylococcus aureus Biofilm Exoprotein Preparation

*S. aureus* CI-182 was streaked onto a 1.5% TSA plate and incubated overnight at 37 °C. A single colony was then resuspended in 0.9% saline to obtain 1 McFarland (McF) units, followed by dilution in tryptic soy broth (TSB) (Oxoid, Thebarton, SA, Australia) at a ratio of 1 in 15. The cultures were then incubated at 37 °C for 48 h in 6-well plates at 70 rpm to form biofilms. The biofilm supernatants were harvested and filtered using a 0.22-µm syringe filter (PALL Acrodisc, New York, NY, USA) to eliminate any planktonic bacteria and bacterial debris and obtain the exoproteins. The exoproteins were concentrated using a 3k MWCO Pierce Protein Concentrator PES (Scoresby, Victoria, Australia) at 3000 rpm and 4 °C to concentrations of 200 μg/mL.

### 4.4. Bradford Protein Assay

The Bradford protein assay (Biorad, Hercules, CA, USA) was performed according to the manufacturer’s instructions to determine the exoprotein concentration. The Bradford protein assay was performed in triplicates for each sample, and the average protein concentration was reported.

### 4.5. Selection and Preparation of S. aureus Clinical Isolates for Inoculation

Two days prior to inoculation, the *S. aureus* clinical isolates, CI-908 and CI-913, were streaked onto 1.5% TSA plates. A single colony was resuspended in 0.9% saline to achieve a concentration of 0.5 McF units, then cultured overnight in TSB at a 1:100 dilution. The pellets from CI-908 and CI-913 were then harvested and resuspended in the exoprotein harvested from CI-182 (as mentioned above) to achieve 2.5 × 10^9^ CFU/mL.

### 4.6. Rhinosinusitis Rat Model

**Figure 3:**
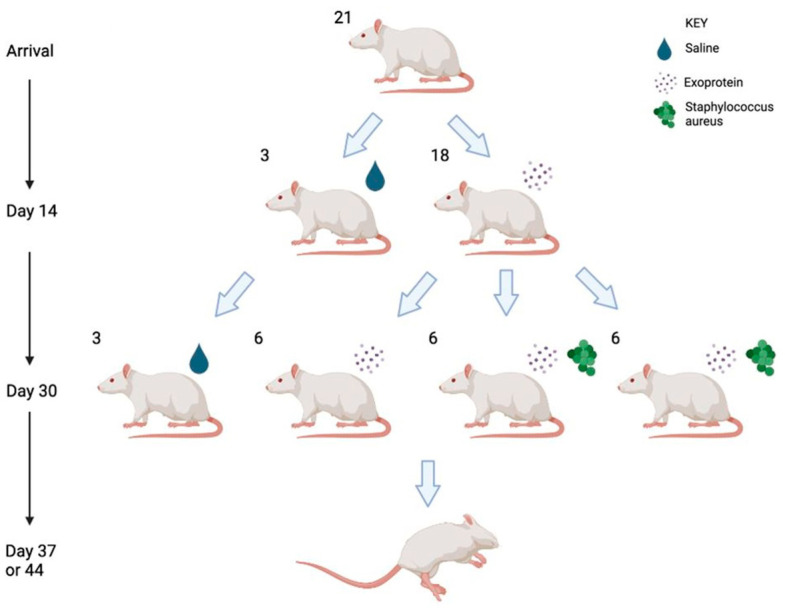
Experimental flowchart.

Sprague Dawley rats (n = 21, all male, 6 weeks of age) were divided to receive once per day, into each nostril, applications of 20 μL of saline for 30 days (group 1, n = 3), 20 μL of 250 μg/mL *S. aureus* CI-182 exoprotein for 30 days (group 2, n = 6), or 20 μL of 250 μg/mL of *S. aureus* CI-182 exoprotein for 13 days (groups 3 and 4), followed by 10^8^ CFU/mL CI-908 (group 3, n = 6) or 10^8^ CFU/mL CI-913 into 20 μL of 250 μg/mL of *S. aureus* CI-182 exoprotein from day 14 to 30 (group 4, n = 6) (Figure 3). The rats were monitored daily, and the intervention was stopped on day 30. The rats were humanely euthanized at two time points, on days 37 and 44 (7 and 14 days after stopping the intervention), respectively. The nasal cavities were rinsed with 200 μL 0.9% saline and collected for establishing the CFUs. The nasal tissues were then harvested and placed in 10% EDTA for decalcification for four weeks. After decalcification, the nasal tissues were fixed in 10% neutral buffer formalin for histopathological examination.

### 4.7. CFU Counts

The nasal rinses were kept on ice, serially diluted with 0.9% saline, and spotted in triplicates on sheep blood agar (Beckton Dickenson, Franklin Lakes, NJ, USA), then incubated at 37 °C overnight. The CFUs were counted and calculated.

### 4.8. Histopathology Examination with Haematoxylin and Eosin and Gram Staining

The decalcified rat heads were embedded in paraffin, and 6 µm sections were cut and stained with haematoxylin and eosin (H&E) or gram staining using standard protocols. Ten areas were selected and graded from 0 to 3 for inflammation for both the anterior and posterior segments in accordance with Houtak et al. (Supplementary Figures S1 and S2) [57].

### 4.9. Genomic DNA Extraction and Sequencing

For the *S. aureus* isolates from the exoprotein group as well as CI-182, CI-908, and CI-913, whole genome sequencing was performed before and after the intervention. The genomic DNA was extracted using the DNeasy Blood and Tissue Kit (Qiagen, 69504, Hilden, Germany) following the manufacturer’s guidelines. The genomic DNA was sequenced using the Oxford Nanopore Technologies (ONT) Gridion Device (Oxford Nanopore Technologies, Oxford, UK). The SQK-RBK 114.96 Rapid Barcoding Kit (Oxford Nanopore Technology) was used with R10.4.1 MinION flowcells (Oxford Nanopore Technology). Base-calling was conducted with Dorado v 0.4.0 in the super accuracy mode, using the ‘r10.4.1 e8.2 400bps_sup@v4.2.0’ configuration (Oxford Nanopore Technology).

### 4.10. Bioinformatics

Adapters and barcodes were removed from long reads using Porechop [58], with long-read-only assemblies created using Flye v2.9.2 with the option “–nano-hq.” [59]. Sequence types were assigned using multi-locus sequence typing (MLST) in the MLST program [60] (Supplementary Table S1).

### 4.11. Statistical Analysis

The statistical analysis of the data was performed using GraphPad Prism 8.0 (GraphPad Software, La Jolla, CA, USA). The statistical significance was determined using a one-way (analysis of variance) ANOVA with Tukey’s multiple comparisons, which was used to test for differences in single independent variables (i.e., inflammation scores and log_10_ CFU among the different treatment groups). Tukey’s multiple comparisons was used as a Tukey test compares the means of one group with every other treatment group. A *p*-value of <0.05 was considered significant.

## 5. Conclusions

Our study successfully establishes the first rat model with a focus on lymphoplasmacytic inflammation in the sinuses. The combination of the *S. aureus* exoprotein with live *S. aureus* bacteria induces inflammation that persists for a minimum of two weeks post-intervention. This model suggests the possible creation of a self-sustaining inflammatory response at least in the short term. Through the effective generation of robust lymphoplasmacytic infiltration, this model opens the door for additional research into this rarer inflammatory subtype. It presents a valuable tool for investigating mechanisms and interventions related to sinusitis in a preclinical setting.

## Figures and Tables

**Table 1:** *S. aureus* clinical isolate patient summary. CI = clinical isolates; yo = years old; GORD = gastro-esophageal reflux disease; CRSwNP = chronic rhinosinusitis with nasal polyps; SNOT 22 = sino-nasal outcome test; LM = Lund–Mackay; LK = Lund–Kennedy. *S. aureus*: *Staphylococcus aureus*.

CI-182	CI-908	CI-913
**Male**	Female	Female
**62 yo**	73 yo	53 yo
**GORD** and **asthma**	Asthma	Asthma and aspirin sensitivity
**CRSwNP**	CRSwNP	CRSwNP
**SNOT 22–34**	SNOT 22–71	SNOT 22–69
**LM: 20**	LM: 20	LM: 24
**LK: 18**	LK: 18	LK: 20

**Table 2:** Inflammatory infiltration scoring. The representative image (×40) was selected to represent each score.

Histology	Grade
** 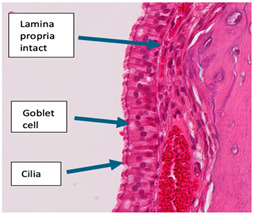 **	Grade 0: Unaffected area of the pseudostratified ciliated columnar epithelium with a normal number of goblet cells and minimal inflammatory infiltration. The lining epithelium was intact.
** 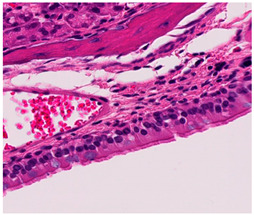 **	Grade 1: Mild lymphoplasmacytic infiltration in the lamina propria.
** 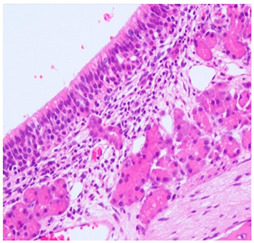 **	Grade 2: Moderate lymphoplasmacytic infiltration in the lamina propria with the lymphocytic infiltration of the epithelium lining, resulting in some disorganization and disruption of the epithelium and loss of cilia.
** 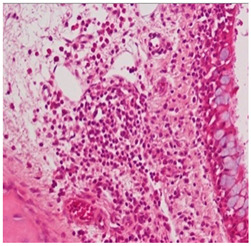 **	Grade 3: Severe lymphoplasmacytic infiltration of the edematous lamina propria. The respiratory epithelium is disrupted and sometimes ulcerated (top right of image), with marked epithelial lymphocytic infiltration and goblet cell hyperplasia.

**Table 3:** Representative inflammation for the control (saline), exoprotein, CI-908, and CI-913-treated rats. There are three representative areas of inflammation for each sample, with a low-powered (10×) view and high-powered (40×) view of each of those areas.

Magnification	Saline	Exoprotein	Staph CI908	Staph CI913
10×#1	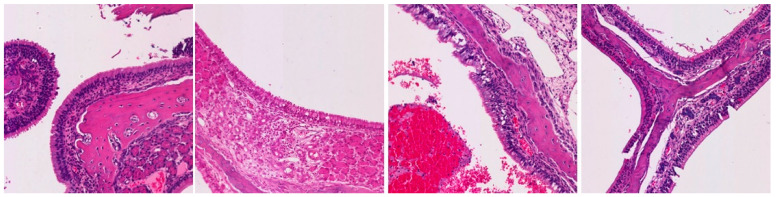			
40×#1	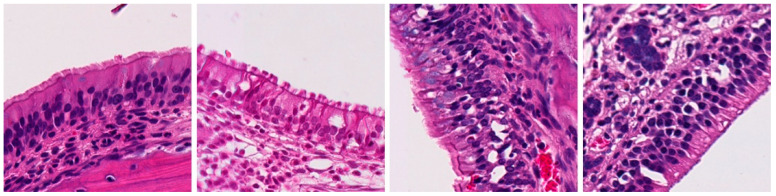			
10×#2	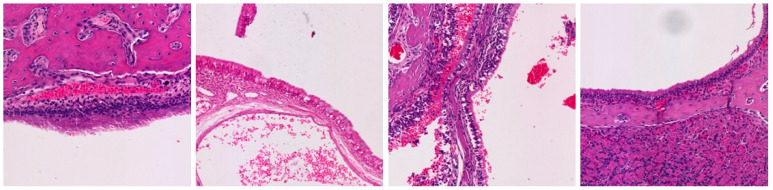			
40×#2	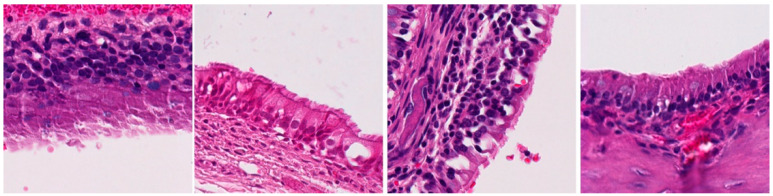			
10×#3	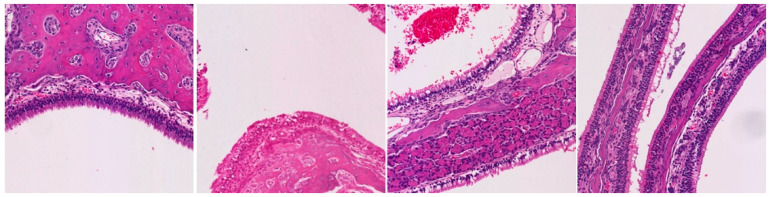			
40×#3	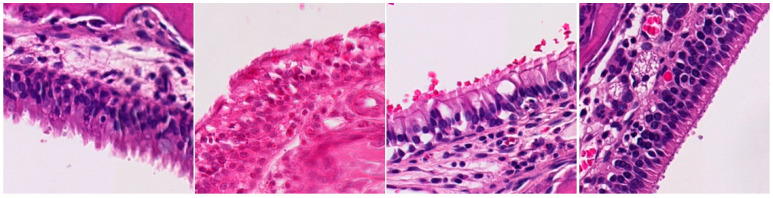			

## Data Availability

The data are contained within the article and Supplementary Materials.

## Supplementary Materials

The supporting information can be downloaded at: https://www.mdpi.com/article/10.3390/ijms25063336/s1.
