# Supplementary material for: A Novel Model of Staphylococcus aureus-Induced Lymphoplasmacytic Rhinosinusitis in Rats
Source: Int J Mol Sci. 2024 Mar 15;25(6):3336. doi: 10.3390/ijms25063336 (PMC10970618; doi:10.3390/ijms25063336)
Supplement: Supplementary file 1 [file ijms-25-03336-s001.zip › ijms-2903475-supplementary.pdf]

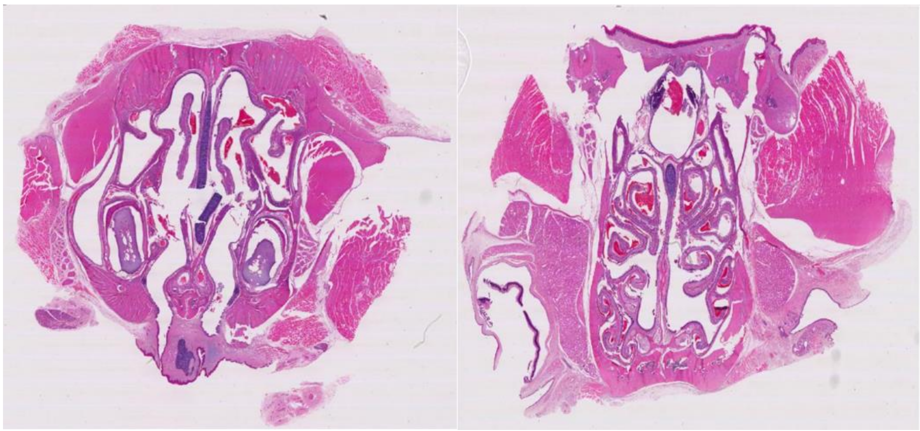

**Supplementary Figure S1. Anterior (Left) and posterior (Right) sections of the rat nasal cavity in H&E staining.**

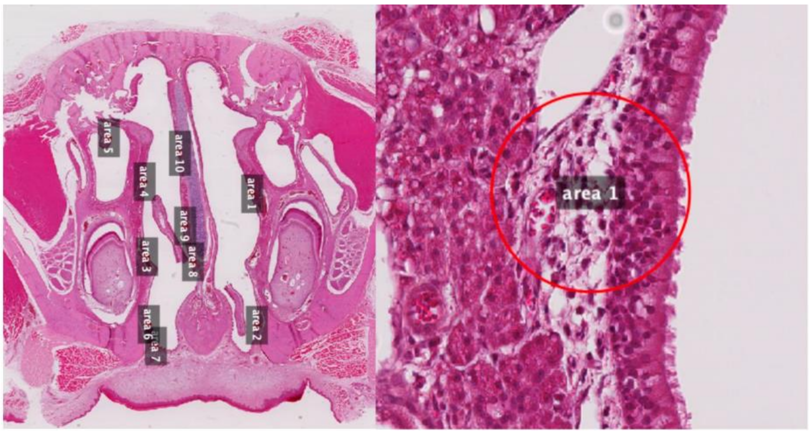


**Supplementary Figure S2. A completely labelled slide of areas of maximal inflammation mapped (Left). A labelled area of maximal infiltrate (Right).**

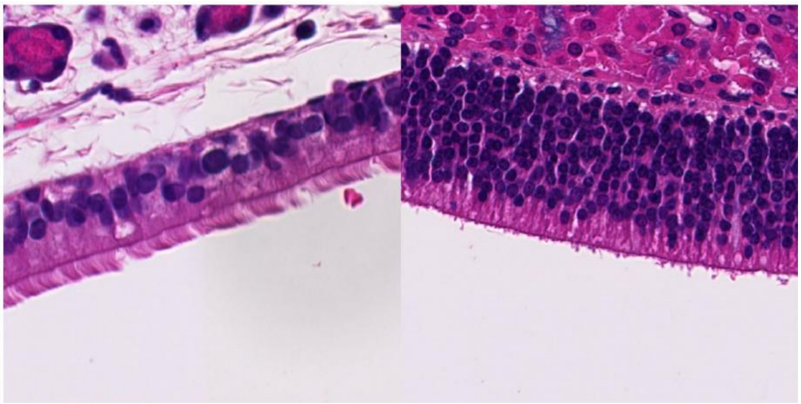

**Supplementary Figure S3. Respiratory epithelium (Left) and olfactory epithelium inflammation (Right)**

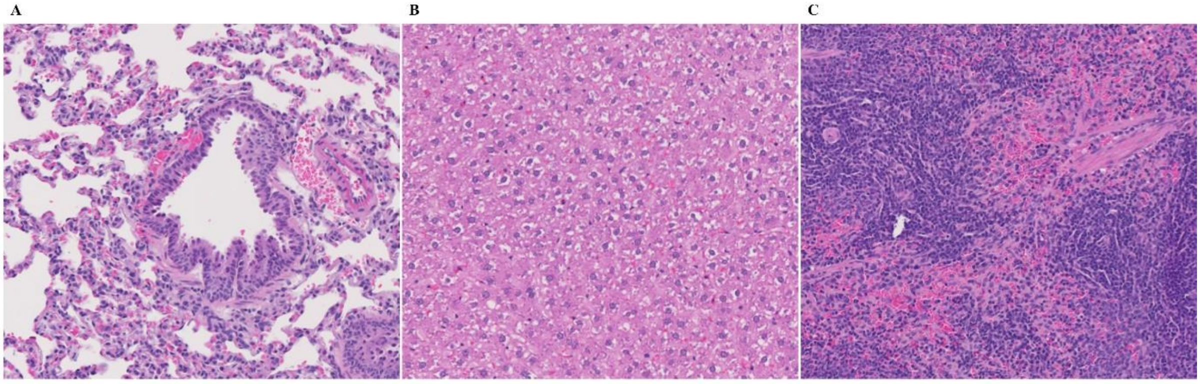


**Supplementary Figure S4. (A) Lung histology with non-specific bronchioalveolar infiltrate. (B) Liver histology. (C) Spleen histology.**

**Supplementary Table S1.** Long read sequences.

| **Group** | **Sequence type** |
| --- | --- |
| Exoprotein | Type 976 |
| CI908 | Type 1290 |
| CI913 | Type 976 |
| CI182 | Type 20 |
